# Multishelled Ni‐Rich Li(Ni*_x_*Co*_y_*Mn*_z_*)O_2_ Hollow Fibers with Low Cation Mixing as High‐Performance Cathode Materials for Li‐Ion Batteries

**DOI:** 10.1002/advs.201600262

**Published:** 2016-09-07

**Authors:** Yihui Zou, Xianfeng Yang, Chunxiao Lv, Tongchao Liu, Yanzhi Xia, Lu Shang, Geoffrey I. N. Waterhouse, Dongjiang Yang, Tierui Zhang

**Affiliations:** ^1^ Collaborative Innovation Center for Marine Biomass FibersMaterials and Textiles of Shandong ProvinceSchool of Environmental Science and EngineeringQingdao UniversityQingdao266071P. R. China; ^2^ Analytical and Testing CentreSouth China University of TechnologyGuangzhou510640China; ^3^ School of Advanced MaterialsPeking University Shenzhen Graduate SchoolPeking UniversityShenzhen518055China; ^4^ Key Laboratory of Photochemical Conversion and Optoelectronic MaterialsTechnical Institute of Physics and ChemistryChinese Academy of SciencesBeijing100190China; ^5^ School of Chemical Sciencesthe University of AucklandAuckland1142New Zealand

**Keywords:** alginate, cathode materials, Li‐ion batteries, Li(Ni*_x_*Co*_y_*Mn*_z_*)O_2_, multishelled hollow fibers

## Abstract

**A simple seaweed biomass conversion strategy** is proposed to synthesize highly porous multishelled Ni‐rich Li(Ni*_x_*Co*_y_*Mn*_z_*)O_2_ hollow fibers with very low cation mixing. The low cation mixing results from the cation confinement by the novel “egg‐box” structure in the alginate template. These hollow fibers exhibit remarkable energy density, high‐rate capacity, and long‐term cycling stability when used as cathode material for Li‐ion batteries.

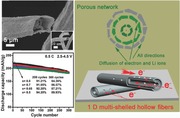

Layered Li(Ni*_x_*Co*_y_*Mn*_z_*)O_2_ is one of the most promising cathode materials for lithium ion batteries (LIBs), due to its stable structure, compositional flexibility, thermal stability, low cost, and relatively high reversible capacity.^1^, ^2^ In particular, the Ni‐rich oxides such as Li(Ni*_x_*Co*_y_*Mn*_z_*)O_2_ (*x* ≥ 0.5) have attracted intense research attention,^3^ since they provide very high specific capacity, ≈212 mAh g^−1^ for *x* = 0.8 and 220 mAh g^−1^ for *x* = 0.86. However, unlike low Ni content oxides which generally exhibit outstanding stabilities,^2^ Ni‐rich oxides suffer inherent drawbacks including poor cycle life and rate performance due to low Li diffusion rates caused by cation mixing. This manifests as Ni^2+^ ions occupying 3b Li sites in the Li slab, whilst Li^+^ ions also occupy sites in the transition metal (TM) layers. This cation mixing leads to a higher activation energy barrier for Li diffusion because of the smaller separations between the TM layers, and also leads to structural instability during electrochemical charge/discharge cycles.^4^ Many approaches have been adopted to address the cation mixing and disorder in Ni‐rich materials. Most approaches focus on adjusting the synthesis conditions to reduce cation migration from the TM site to Li site by controlling the lithiation temperature^5^ or the Li/TM ratio.^6^ However, in these methods, an excess of Li is necessary to produce highly ordered Ni‐rich oxides. Residual Li remains on the surface of the active materials and reacts with air to form LiOH and Li_2_CO_3_, leading to undesirable side reactions with the electrolyte. The undesirable LiOH and Li_2_CO_3_ species also impede the diffusion of Li^+^ ions due to their insulating properties, and thus deteriorate the electrochemical cycle performance.^5^ In addition, traditional synthesis methods, which usually involve coprecipitation and annealing at high temperature, do not allow controllable synthesis of Ni‐rich nanostructures needed to address the sluggish Li^+^ ion diffusion.^6^, ^7^ Therefore, excellent rate performance and high specific capacity are hard to achieve.

Structure can strongly influence the functionality of electrode materials. To avoid cation mixing and thus improve the performance of Ni‐rich Li(Ni*_x_*Co*_y_*Mn*_z_*)O_2_ electrodes, the design of 1D structures is a prudent strategy. 1D structures also guarantee a high electrode–electrolyte contact area and a short transport path for electrons and Li^+^ ions,^8^ all of which could be expected to improve the rate performance of the Li(Ni*_x_*Co*_y_*Mn*_z_*)O_2_ materials. In addition, porous electrodes with multishelled structures have attracted interest in recent years based on the following advantages.^9^ First, porous multishelled structures offer more channels for Li^+^ ions and thus are helpful for enhancing the specific capacity. Second, the multishelled structures have a very short path for Li^+^ ion diffusion, leading to good rate performance. Thus, it is easily envisaged that a multishelled Ni‐rich Li(Ni*_x_*Co*_y_*Mn*_z_*)O_2_ hollow fiber (HF), combining the advantages of a 1D morphology and a porous multishelled structure, should exhibit high‐performance as cathode materials for LIBs.

In this Communication, we report the synthesis of a series of multishelled Ni‐rich Li(Ni*_x_*Co*_y_*Mn*_z_*)O_2_ (*x* = 0.8, 0.7, 0.65, and 0.5) HFs with low cation mixing using sustainable seaweed (alginate) fiber as template. The M^n+^(M = Ni, Co, Mn) cations were first immobilized into a novel “egg‐box” arrangement via coordination with negatively charged α‐l‐guluronate (G) blocks of the linear alginate macromolecule.^10^ Meanwhile, the β‐d‐mannuronate (M) blocks in alginate can absorb Li^+^ via the negatively charged carboxyl groups.^11^ This approach suppresses cation mixing or the formation of undesirable LiOH and Li*_x_*CO_3_ species in subsequent calcination step used to synthesize the HFs. The Li‐M alginate fibers (Li‐M‐AFs) were converted to multishelled HFs by calcination. The combustion of the alginate precursor introduces high porosity in the fibers. As expected, the 1D multishelled Li(Ni*_x_*Co*_y_*Mn*_z_*)O_2_ HFs demonstrate superior discharge capacity of 219.9 mAh g^−1^ at 0.5 C (*x* = 0.8) compared with conventional Li(Ni*_x_*Co*_y_*Mn*_z_*)O_2_ materials, and an outstanding capacity retention of 84.36% after 300 cycles.

The synthesis of the multishelled Ni‐rich Li(Ni*_x_*Co*_y_*Mn*_z_*)O_2_ HFs is described in **Scheme** **1**a. Calcium alginate microfibers (Ca‐AFs) were prepared from aqueous sodium alginate using a wet‐spinning method (see Synthesis of multi‐shelled Li(Ni*_x_*Co*_y_*Mn*_z_*)O_2_ hollow fiber, Supporting Information), and used as templates. The obtained Ca‐AFs were then transformed into protonated alginate fibers (H‐AFs) by immersion in a 1 m HCl aqueous, resulting in complete Ca^2+^/H^+^ exchange. The protonated alginate was then soaked in a mixed aqueous solution of cobalt acetate, nickel acetate, and manganese acetate to form purple M‐alginate fibers (M‐AFs). This immobilized the M^n+^ cations (M = Ni, Co, Mn, respectively) into an “egg‐box” arrangement through coordination by the four G‐block of alginate.^12^, ^13^ The molar ratio of Ni/Co/Mn in the M‐AFs can be easily controlled by adjusting the ratio of the three cations in the aqueous solution. Herein, four different M‐AFs were prepared with precisely controlled Ni/Co/Mn molar ratios (see Table S1, Supporting Information). The M‐AFs were then dispersed in a suspension of Li_2_CO_3_ in H_2_O:EtOH (1:2) for 0.5 h to obtain the Li‐M‐AFs, in which the Li^+^ was immobilized electrostatically by the alginate carboxyl groups.

**Scheme 1:**
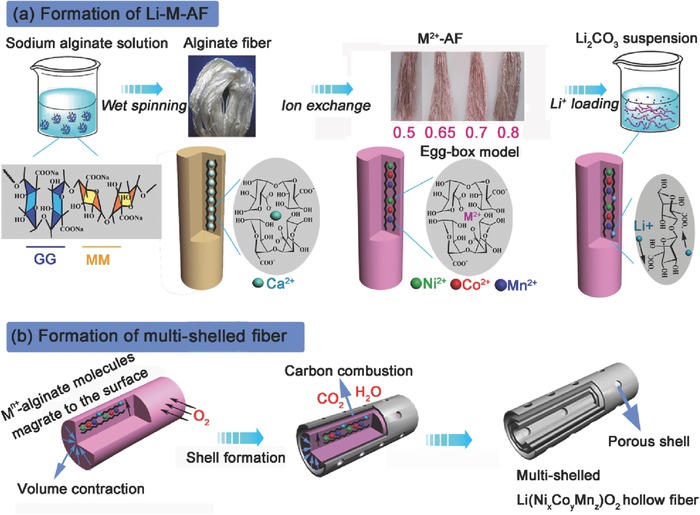
Procedure for the fabrication of multishelled Li(Ni*_x_*Co*_y_*Mn*_z_*)O_2_ hollow fibers from sustainable alginate fibers.

Calcination of the Li‐M‐AFs at different temperatures yielded multishelled Li(Ni*_x_*Co*_y_*Mn*_z_*)O_2_ HFs (*x* = 0.8, 0.7, 0.65, and 0.5). The morphology and structure of Li(Ni*_x_*Co*_y_*Mn*_z_*)O_2_ HFs were characterized by field emission scanning electron microscopy (FESEM). As shown in **Figure** **1**, the five Li(Ni*_x_*Co*_y_*Mn*_z_*)O_2_ samples exhibit a 1D fibrous morphology. The diameter of these fibers is ≈10 μm, which is approximately one half that of the Ca‐AFs (see Figure S1, Supporting Information, ≈20 μm) and the ion‐exchanged Li‐M‐AFs (see Figure S2, Supporting Information, ≈20 μm). The shrinkage of the fibers during the calcination step can mainly be attributed to combustion of the alginate network in the fibers. Interestingly, the cross‐section images of the fibers reveal a novel multishelled hollow structure (see Figure 1a–d). Most of the fibers are highly porous with double or triple shells (see Figure S3, Supporting Information), with the thickness of the individual shells reaching several hundred nanometers. The multishelled morphology with wide space between the shells is highly desirable, as it is expected to effectively increase the electrode/electrolyte contact area and enhance the diffusion rates for both Li^+^ ions and electrons. Elemental mapping by energy dispersive X‐ray spectroscopy (EDS) was used to analyze the composition of Li(Ni_0.65_Co_0.25_Mn_0.1_)O_2_ HFs. As shown in Figure 1e, Ni, Co, and Mn are homogeneously distributed over a single fiber. The results from the EDS analysis (see Figure 1f) reveal that the molar ratios of Ni, Co, and Mn in the fibers were in excellent accord with the nominal values of 0.65:0.25:0.1. The accurate chemical composition of the fibers was determined using inductive coupled plasma atomic emission spectrometry (ICP‐AES). Excellent agreement was found between the nominal and actual molar ratios of Li, Ni, Co, Mn in the Li(Ni*_x_*Co*_y_*Mn*_z_*)O_2_ HFs (see Table S2, Supporting Information).

**Figure 1:**
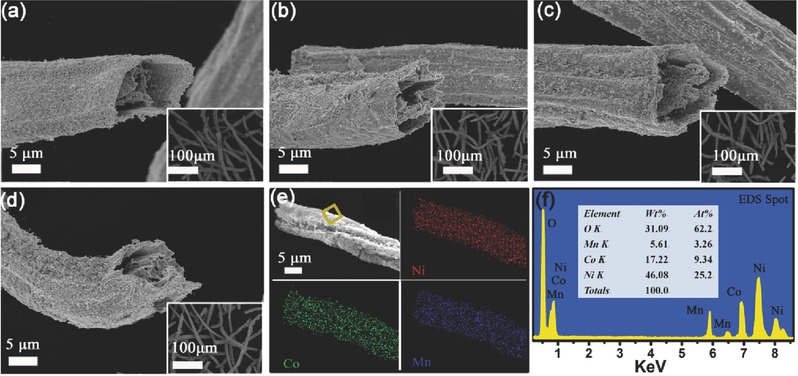
Cross‐section SEM images of the multishelled Li(Ni*_x_*Co*_y_*Mn*_z_*)O_2_ hollow fibers a) *x* = 0.8, b) *x* = 0.7, c) *x* = 0.65, d) *x* = 0.5. Insets: the aspect ratio of the fibers at lower magnification. e) SEM image of Li(Ni_0.65_Co_0.25_Mn_0.1_)O_2_ and the corresponding EDS mapping for Ni, Co, Mn elements. f) EDS spectrum collected from the area in panel (e) of Li(Ni_0.65_Co_0.25_Mn_0.1_)O_2_.

The information above allows the multishelled structure of the Li(Ni*_x_*Co*_y_*Mn*_z_*)O_2_ HFs to be rationalized, as illustrated in Scheme 1b. In the initial stage of the calcination process, the long‐chain M^n+^‐alginate molecules partially migrate toward the surface of the fibers, whilst the inner solid core contracts to form a core–shell structure due to nonequilibrium heat transfer. With heating to higher temperatures, the migration of M^n+^‐alginate molecules and inner core contraction reoccurs, along with combustion of the alginate template resulting in the formation of the porous multishelled Li(Ni*_x_*Co*_y_*Mn*_z_*)O_2_ HFs. FESEM images of Li‐M‐AFs heated from 250 to 750 °C successfully document the evolution of the porous multishelled hollow structure (see Figure S4, Supporting Information). The first shell appears at temperature around 450 °C, and the porous network and the second shell appear around 550 °C. Complete combustion of alginate‐derived carbonaceous component yields Li(Ni*_x_*Co*_y_*Mn*_z_*)O_2_ shells. The carbon combustion creates voids in the shells, and thus the multishelled HFs are highly porous. The space between the shells ensures excellent electrode–electrolyte contact, whilst the porous network in the shells provides a short pathway for electron and Li^+^ diffusion, both of which could be expected to significantly improve the rate performance and the specific capacity of Li(Ni*_x_*Co*_y_*Mn*_z_*)O_2_.

Powder X‐ray diffraction (XRD) patterns for all the Li(Ni*_x_*Co*_y_*Mn*_z_*)O_2_ HFs are shown in Figure S5 of the Supporting Information. The (006)/(102) and (108)/(110) doublets indicate that all Li(Ni*_x_*Co*_y_*Mn*_z_*)O_2_ HFs possess a well‐defined layered structure based on a hexagonal a‐NaFeO_2_ structure with a *R* $\bar{3}m\;$space group.^14^ The ratio *I*_(003)_/*I*_(104)_ reflects the degree of cation mixing caused by occupation of Li^+^ sites by Ni^2+^, which occurs due to the similar ionic radii of Ni^2+^(0.069 nm) and Li^+^(0.076 nm).^15^ Cation mixing seriously weakens cycle life and rate performance of Li(Ni*_x_*Co*_y_*Mn*_z_*)O_2_ materials. As shown in Table S3 of the Supporting Information, the ratio of *I*_(003)_/*I*_(104)_ for the Ni‐rich HFs ranges from 1.31 for *x* = 0.5 to 1.24 for *x* = 0.8. Given that the cation mixing is considered negligible if the ratio *I*_(003)_/*I*_(104)_ is higher than 1.20,^16^ it can be concluded that the Ni‐rich Li(Ni*_x_*Co*_y_*Mn*_z_*)O_2_ HFs synthesized here exhibit an unusually low degree of cation mixing. To probe Li^+^/Ni^2+^ ion disorder in detail, we refined the XRD patterns based on the Rietveld method using General Structure Analysis System (GSAS) software (**Figure** **2**a). The initial occupation parameters of all atoms are based on formula (Li_1_Ni_2_)_3b_(Li_2_NiCo_1_Mn_1_)_3a_O_2_, in which Li_1_/Ni_2_are set at 3b site, Li_2_/Ni_1_/Co_1_/Mn_1_ at 3a site, and O at 6c site. The occupation parameters of Co and Mn at the 3a site are fixed, and the total amount of Li and Ni within the materials fixed, while the distribution of Li and Ni between the 3a and 3b sites is variable. According to the calculated occupation parameters, the crystallographic formulas for the multishelled Li(Ni*_x_*Co*_y_*Mn*_z_*)O_2_ hollow fibers (*x* = 0.8, 0.7, 0.65, and 0.5) are (Li_0.9352_Ni_0.0675_)_3b_(Li_0.0675_Ni_0.7325_Co_0.1_Mn_0.1_)_3a_O_2_, (Li_0.9338_Ni_0.0662_)_3b_(Li_0.0662_Ni_0.6338_Co_0.20_Mn_0.1_)_3a_O_2_, (Li_0.9472_Ni_0.0528_)_3b_(Li_0.0528_Ni_0.5972_Co_0.25_Mn_0.1_)_3a_O_2_, and (Li_0.9515_Ni_0.0485_)_3b_(Li_0.0485_Ni_0.4515_Co_0.2_Mn_0.3_)_3a_O_2_, respectively. These models provided an excellent fit to the experimental XRD data, as can be seen in Figure 2b–e. With an increase in the Ni content from *x* = 0.5 to 0.8, the Li/Ni exchange increases from 0.0485 to 0.0675, though the latter value is still significantly lower than the value of 0.085 reported for bulk Li(Ni_0.65_Co_0.25_Mn_0.1_)O_2_ with *x* = 0.7.^2^ The XRD patterns of the Li(Ni_0.65_Co_0.25_Mn_0.1_)O_2_ precursor calcined at temperatures from 150 to 750 °C are shown in Figure S6 of the Supporting Information. Crystalline Li(Ni_0.65_Co_0.25_Mn_0.1_)O_2_ appears when the annealing temperature is higher than 350 °C. The ratio *I*_(003)_/*I*_(104)_ is higher than 1.26 at calcination temperatures above 350 °C (see Table S4, Supporting Information). At lower temperatures, the M^2+^ ions are confined into the “egg‐box” and cannot migrate freely to Li^+^ sites (see Scheme 1a). At high temperatures, the “egg‐box” converts to carbon/metal core/shell structure,^10^, ^11^, ^12^ with the carbon shell preventing cation mixing until it is completely consumed by combustion. Obviously, the use of the Li‐M‐AF precursor plays a key role in minimizing cation disorder during the calcination step used in the synthesis of the Li(Ni_0.65_Co_0.25_Mn_0.1_)O_2_ HFs.

**Figure 2:**
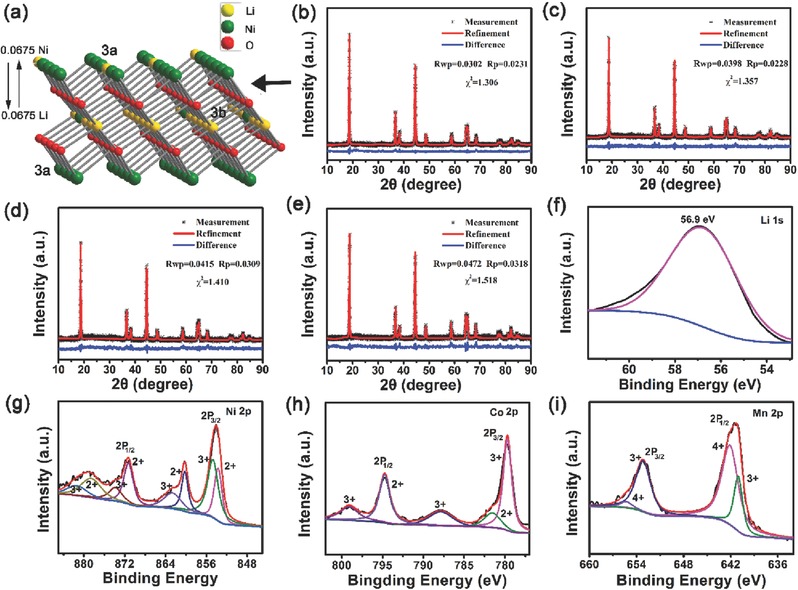
a) Structure model for Li(Ni*_x_*Co*_y_*Mn*_z_*)O_2_ with *x* = 0.8, b–e) Rietveld refinement of XRD patterns for Li(Ni*_x_*Co*_y_*Mn*_z_*)O_2_ for b) *x* = 0.8, c) *x* = 0.7, d) *x* = 0.65, e) *x* = 0.5. f–i) XPS spectra for Li(Ni_0.65_Co_0.25_Mn_0.1_)O_2_. (f) Li 1s, (g) Ni 2p, (h) Co 2p, and (i) Mn 2p.

X‐ray photoelectron spectroscopy (XPS) examines the valence states of Li, Ni, Co, Mn, and O in the Li(Ni*_x_*Co*_y_*Mn*_z_*)O_2_ HFs. Li 1s, Ni 2p, Co 2p, Mn 2p, and O 1s XPS spectra for Li(Ni_0.65_Co_0.25_Mn_0.1_)O_2_, which exhibits the best cycle performance in the electrochemical tests, are displayed in Figure 2. The corresponding XPS survey spectrum is shown in Figure S7a of the Supporting Information. The spectra presented here for Li(Ni_0.65_Co_0.25_Mn_0.1_)O_2_ are representative of all the Li(Ni*_x_*Co*_y_*Mn*_z_*)O_2_ HFs synthesized in this work. The C 1s peak is due to the residual hydrocarbon on the sample surface during the synthesis process. Deconvolution of Li 1s in Figure 2f gives the peak with binding energies of 56.9 eV, which is in good agreement with the results reported in lithium oxide cathode materials.^17^ The Ni 2p_3/2_ peak at 854.2 eV and associated satellite feature at 861.2 eV are characteristic for a Ni(II) oxide species (Figure 2g).^18^ The Ni 2p_3/2_ peak at 855.8 eV indicates the coexistence of Ni(III).^19^ The Co2p_3/2_ region contained two peaks (Figure 2h), a main peak at 779.0 eV and the weaker peak at 780.4 eV which are assigned to tri‐ and divalent Co species,^1^, ^20^ respectively. The Mn 2p_3/2_ peak was deconvoluted into two signals at 641.7 and 642.5 eV, which are assigned to Mn(III) and Mn(IV) species, respectively.[[qv: 1e]] The O1s spectrum (Figure S7b, Supporting Information) contains three signals peaks at 526.6, 528.9, and 530.3 eV, assigned to coordinated oxygen, lattice oxygen, and coordinatively unsaturated surface oxygen,^21^ respectively. Surface carbonate may also contribute to the O 1s spectrum, since a C 1s signal was seen in the survey spectrum and assigned to a carbonate species. In any case, the XPS results are in good agreement with the previously reported Ni‐rich (*x* = 0.65) Li(Ni*_x_*Co*_y_*Mn*_z_*)O_2_ materials.^19^

Representative transmission electron microscopy (TEM) and high‐resolution TEM (HRTEM) images of Li(Ni_0.65_Co_0.25_Mn_0.1_)O_2_ HFs are shown in **Figure** **3**. Figure 3a shows a fragment of the porous HF shell, and is composed of nanoparticles with a mean size of ≈120 nm. The corresponding selected area electron diffraction (SAED) pattern (Figure 3b) reveals the shell is polycrystalline. Figure 3c shows a single‐crystal SAED pattern taken from one of the nanoparticles, which can be indexed to hexagonal Li(Ni_0.65_Co_0.25_Mn_0.1_)O_2_ projected alone its [100] zone axis. A HRTEM image was taken from the nanoparticle facet boxed in Figure 3a, and is shown in Figure 3b. The HRTEM image shows *d*‐spacings of 0.23 and 0.47 nm, corresponding to (012) and (003) lattice fringes of hexagonal Li(Ni_0.65_Co_0.25_Mn_0.1_)O_2_ (space group *R* $\bar{3}m$, *a* = *b* = 0.2873, and *c* = 1.4273 nm). The corresponding fast Fourier transform (FFT) pattern (inset in Figure 1d) is equivalent to the SAED pattern of the whole particle.

**Figure 3:**
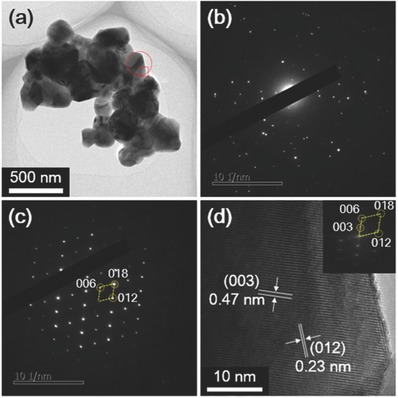
a) TEM image of a typical fragment of a Li(Ni_0.65_Co_0.25_Mn_0.1_)O_2_ HF, b) SAED pattern of the whole fragment, c) SAED pattern of the circled area in (a), d) HRTEM image of the square area in (a) with the corresponding FFT pattern (inset).

To evaluate the potential of the multishelled Li(Ni*_x_*Co*_y_*Mn*_z_*)O_2_ HFs as the cathode materials for LIBs, their electrochemical properties were examined by cyclic voltammetry (CV) and galvanostatic cycling techniques. The initial charge/discharge curves, obtained from cells containing multishelled Li(Ni*_x_*Co*_y_*Mn*_z_*)O_2_ HFs (*x* = 0.8, 0.7, 0.65, and 0.5) cycling between 2.5 and 4.5 V at a rate of 20 mA g^−1^ at 25 °C, are presented in **Figure** **4**a. The initial discharge capacity of the electrodes increased with increasing Ni content (*x*), which can likely be attributed to the lower diffusion energy barriers to delithiation of Ni‐sites.^22^ The discharge capacities are 229.9, 217.2, 211.5, and 204.4 mAh g^−1^ for *x* = 0.8, 0.7, 0.65, and 0.5, respectively. These values are among the best yet reported Li(Ni*_x_*Co*_y_*Mn*_z_*)O_2_ cathodes. The gap between the theoretical (≈275 mAh g^−1^) and observed capacities is ascribed to irreversible reactions, such as the decomposition of the electrolyte.^23^ CVs for all electrode materials are shown in Figure S8, Supporting Information. The Li(Ni*_x_*Co*_y_*Mn*_z_*)O_2_ samples show peaks at 3.76 V on charging and 3.72 V on discharging. A further higher charging potential peak at about 4.2 V is seen for cells containing Ni‐rich Li/Li(Ni*_x_*Co*_y_*Mn*_z_*)O_2_ HFs (*x* = 0.8 and 0.7). The two delithiation stages at 3.76 V and 4.2 V likely correspond to the oxidation of Ni^2+^ to Ni^3+^ and Ni^3+^ to Ni^4+^, respectively, in such Ni‐rich samples.^22^, ^24^

**Figure 4:**
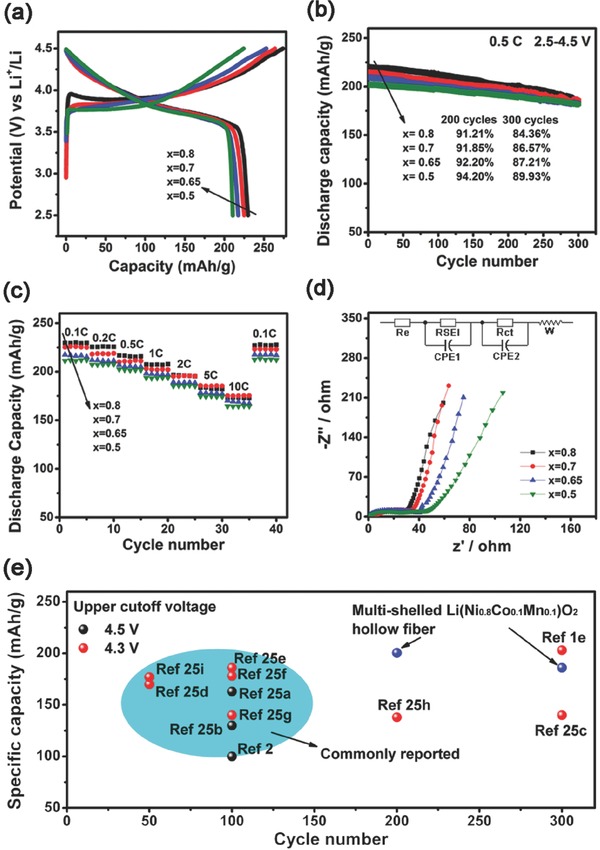
a) Initial charge–discharge curves at a rate of 0.1 C (20 mA g^−1^) between 2.5 and 4.5 V, b) cycle performance, c) rate performance, d) Nyquist plots of the multishelled Li(Ni*_x_*Co*_y_*Mn*_z_*)O_2_ HFs (*x* = 0.8, 0.7, 0.65, and 0.5), and e) comparison of specific capacity and cycle life between multishelled Li(Ni_0.8_Co_0.1_Mn_0.1_)O_2_ electrodes and various recently reported high‐performance Li(Ni_0.8_Co_0.1_Mn_0.1_)O_2_ electrodes.

Figure 4b compares the cycling performance of the multishelled Li(Ni*_x_*Co*_y_*Mn*_z_*)O_2_ HFs. After 200 deep discharge/charge cycles between 2.5–4.5 V at 0.5 C, the samples still retain discharge capacities of 200.4, 197.0, 193.1, and 190.5 mAh g^−1^, for *x* = 0.8, 0.7, 0.65 and 0.5, respectively. Capacity retentions are therefore 91.21%, 91.85%, 92.20%, and 94.20%, respectively. After 300 electrochemical cycles, the capacity retention was 84.36% for *x* = 0.8. This performance level is far superior to most Ni‐rich electrodes reported (Figure 4e).^1^, ^2^, ^25^ Even when the Li/Li(Ni*_x_*Co*_y_*Mn*_z_*)O_2_ cells were tested at a high cut‐off potential of 4.6 V versus Li/Li^+^ for 100 cycles (Figure S9, Supporting Information), the capacity retention for the electrode with *x* = 0.5 was still 91.40%, much higher than the best previously reported value of 85%.^12^ Clearly, the multishelled hollow fibrous electrodes exhibit outstanding cycling stability, compared with the convention Ni‐rich Li(Ni*_x_*Co*_y_*Mn*_z_*)O_2_ electrodes.^14^

Extended cycling stability measurements were conducted at different current densities. As shown in Figure 4c, all the Ni‐rich Li(Ni*_x_*Co*_y_*Mn*_z_*)O_2_ electrodes exhibit excellent rate capability on increasing the current density stepwise from 0.1 to 10 C. Example, as the current density was increased stepwise from 0.1 to 0.2, 0.5, 1, 2, 5, and 10 C, the Li(Ni_0.8_Co_0.1_Mn_0.1_)O_2_ electrode delivered stable capacities of 229.6, 225.5, 216.7, 207.4, 196.6, 183.4, and 172.7 mAh g^−1^, respectively. More importantly, when the current density was returned back to 0.1 C, full capacities were recovered for all the multishelled hollow electrodes, indicating their excellent rate performance. Figure 4d displays impedance spectra for the multishelled Li(Ni*_x_*Co*_y_*Mn*_z_*)O_2_ hollow fibrous electrodes after 5 cycles. The impedance parameters are fitted by the same equivalent circuit (see inset in Figure 4d). The impedance parameters are fitted by the same equivalent circuit shown as the inset in Figure 4d. The high‐frequency intercept at the *Z*′axis is the combined resistance of the electrolyte and cell components (*R*_e_). The high‐middle frequency semicircle is the contribution of the solid electrolyte interface resistance (RSEI) and the charge‐transfer resistance (*R*_ct_) at the interface between the electrolyte and the electrode. The low‐frequency oblique line represents the Warburg impedance (W), which belongs to the Li^+^ ion diffusion process in the electrode materials. At the same cycle, the lowest *R*_ct_ value (31.2 Ω) was found for the Li(Ni_0.8_Co_0.1_Mn_0.1_)O_2_ electrode, indicating that sample possessed the lowest resistance to charge transfer and fastest Li‐intercalation kinetics. Accordingly, the sample possessed the highest discharge capacity amongst the four HF samples tested. Charge transfer resistance was in inversely proportional to the Ni content in the samples, which can be rationalized in terms of the higher delithiation barriers of Co‐ and Mn‐sites in comparison with Ni‐sites.^24^

**Figure** **5** illustrates the advantages of the multishelled Li(Ni*_x_*Co*_y_*Mn*_z_*)O_2_ HFs during electrochemical cycling. First, the 1D morphology of the fiber shells allows fast Li^+^ and e^−^ transport, which is crucial for promoting electrochemical performance. Second, the space between the shells can store the electrolyte by acting as “reservoirs”, there by shortening the diffusion distance of Li^+^ and promoting rapid charge‐transfer. Finally, the porous network on the shell serves to reduce the electron and Li^+^ ion diffusion path, and also enables electrons from all directions to reach the Li(Ni*_x_*Co*_y_*Mn*_z_*)O_2_ particles, which is highly beneficial for improving the specific capacity.^17^ The combination of all these factors contributes to the remarkable cyclability of the multishelled Ni‐rich Li(Ni*_x_*Co*_y_*Mn*_z_*)O_2_ HFs. In order to investigate the fast Li^+^ diffusion in the multishelled Ni‐rich Li(Ni*_x_*Co*_y_*Mn*_z_*)O_2_ HFs, the Li^+^ ion diffusion coefficient was determined via CV analyses (Figure S10, Supporting Information). As the Ni content increased, the Li^+^ ion diffusion coefficient increased, since the delithiation barrier near Ni^2+^/Ni^3+^ is lower compared to that of Co^3+^ or Mn^4+^.^24^ Figure S11 of the Supporting Information also compares the Li^+^ ion diffusion coefficient of commercial bulk‐Li(Ni_0.5_Co_0.2_Mn_0.3_)O_2_ particles (≈10 μm, see Figure S12, Supporting Information) and multishelled Li(Ni_0.5_Co_0.2_Mn_0.3_)O_2_ HFs. The Li^+^ ion diffusion coefficient for multishelled Li(Ni_0.5_Co_0.2_Mn_0.3_)O_2_ HF for delithiationis 2.74 × 10^−8^ cm^2^ S^−1^, much higher than the 1.40 × 10^−8^ cm^2^ S^−1^ determined for bulk‐Li(Ni_0.5_Co_0.2_Mn_0.3_)O_2_. The Li^+^ ion diffusion coefficients of bulk‐Li(Ni_0.5_Co_0.2_Mn_0.3_)O_2_ particles and multishelled Li(Ni_0.5_Co_0.2_Mn_0.3_)O_2_ HF were also measured by using galvanostatic intermittent titration technique (GITT) method. Their GITT data are shown in Figure 5b and Figure S13 and S14 of the Supporting Information, respectively. Figure S15 of the Supporting Information shows the calculated diffusion coefficients at different state of charge (SOC = 0.2, 0.3, 0.4, and 0.5), where the multishelled Li(Ni_0.5_Co_0.2_Mn_0.3_)O_2_ HF and commercial bulk‐Li(Ni_0.5_Co_0.2_Mn_0.3_)O_2_ particles show the same upward trend with the increase of SOC value from 0.2 to 0.5. During the early stage of delithiation, the Li slab space expands to facilitate faster Li^+^ ion diffusion due to the removal of O^2−^–Li^+^–O^2−^ bonds across the slab, and the valence of Ni rises from Ni^2+^ to Ni^3+^ and Ni^4+^, which leads to the increase of diffusion coefficient by reducing effective diffusion barriers.^24^ When SOC = 0.5, the Li^+^ diffusion coefficient of multishelled Li(Ni_0.5_Co_0.2_Mn_0.3_)O_2_ HF is 1.15 × 10^−9^ cm^2^ S^−1^, which is much larger than that of bulk‐Li(Ni_0.5_Co_0.2_Mn_0.3_)O_2_ particles (2 × 10^−10^ cm^2^ S^−1^, see Figure S15, Supporting Information).

**Figure 5:**
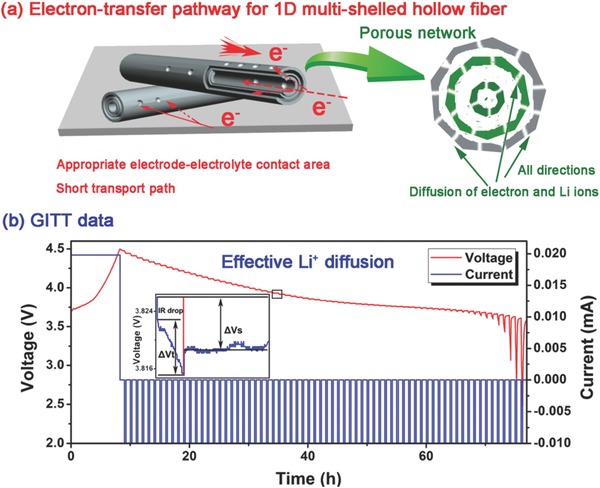
a) Schematic illustration of the advantages of the multishelled Li(Ni*_x_*Co*_y_*Mn*_z_*)O_2_ HFs for lithium battery applications. b) Data from the GITT experiment on multishelled Li(Ni_0.5_Co_0.2_Mn_0.3_)O_2_ HFs.

In summary, a series of multishelled (double or triple) Ni‐rich Li(Ni*_x_*Co*_y_*Mn*_z_*)O_2_ HFs were successfully synthesized by using a sustainable biomass feedstock (sodium alginate) as a template. The novel egg‐box structure of the alginate effectively immobilizes Ni^2+^, Co^2+^, and Mn^2+^ cations, which suppresses cation mixing during a subsequent calcination step used in the synthesis of the Li(Ni*_x_*Co*_y_*Mn*_z_*)O_2_ HFs. These HF cathode materials exhibit much higher specific capacity, better cycling performance and rate capability compared with almost all Ni‐rich Li(Ni*_x_*Co*_y_*Mn*_z_*)O_2_ cathodes reported to date. The very low cation mixing and unique structure characteristics such as 1D morphology and porous multishelled hollow structure synergetically contribute to the outstanding electrochemical performance. This work highlights the potential of multishelled Ni‐rich Li(Ni*_x_*Co*_y_*Mn*_z_*)O_2_ HFs as cathode materials for future lithium ion battery systems.

## Supporting information

As a service to our authors and readers, this journal provides supporting information supplied by the authors. Such materials are peer reviewed and may be re‐organized for online delivery, but are not copy‐edited or typeset. Technical support issues arising from supporting information (other than missing files) should be addressed to the authors.

Supplementary

## References

[1] a) M.‐H. Han , E. Gonzalo , G. Singh , T. Rojo , Energy Environ. Sci. 2015, 8, 81;

[2] C. Fu , G. Luo , D. Li , Q. Fan , J. Li , ACS Appl. Mater. Interfaces 2014, 6, 15822. 25203668 10.1021/am5030726

[3] a) Z.‐J. Wu , D. Wang , Z.‐F. Gao , H.‐F. Yue , W.‐M. Liu , Dalton Trans. 2015, 44, 18624; 26448326 10.1039/c5dt02552d

[4] a) W. Liu , P. G. Oh , X. Liu , M.‐J. Lee , W. Cho , S. J. Chae , Y. Kim , J. Cho , Angew. Chem. Int. Ed. 2015, 54, 4440; 10.1002/anie.201409262 25801735

[5] M. Dixit , M. Kosa , O. S. Lavi , B. Markovsky , D. Aurbach , D. T. Major , Phys. Chem. Chem. Phys. 2016, 18, 6799. 26878345 10.1039/c5cp07128c

[6] a) F. Wu , J. Tian , Y. Su , J. Wang , C. Zhang , L. Bao , T. He , J. Li , S. Chen , ACS Appl. Mater. Interfaces 2015, 7, 7702; 25811905 10.1021/acsami.5b00645

[7] S. Bauer , L. D. Biasi , S. Glatthaar , L. Toukam , H. Gebwein , T. Baumbach , Phys. Chem. Chem. Phys. 2015, 17, 16388. 26051380 10.1039/c5cp02075a

[8] a) M.‐H. Park , M. G. Kim , J. Joo , K. Kim , J. Kim , S. Ahn , Y. Cui , J. Cho , Nano Lett. 2009, 9, 3844; 19746961 10.1021/nl902058c

[9] a) J. Wang , N. Yang , H. Tang , Z. Dong , Q. Jin , M. Yang , D. Kisailus , H. Zhao , Z. Tang , D. Wang , Angew. Chem. Int. Ed. 2013, 52, 6417; 10.1002/anie.201301622 23649876

[10] D. Li , D. Yang , X. Zhu , D. Jing , Y. Xia , Q. Ji , R. Cai , H. Li , Y. Che , J. Mater. Chem. A 2014, 2, 18761.

[11] B. Wang , W. A. Abdulla , D. Wang , X. S. Zhao , Energy Environ. Sci. 2015, 8, 869.

[12] C. X. Lv , X. F. Yang , A. U , Y. Z. Xia , Y. Jia , L. Shang , T. R. Zhang , D. J. Yang , J. Mater. Chem. A 2015, 3, 22708.

[13] L. Liu , X. Yang , N. Ma , H. Liu , Y. Xia , C. Chen , D. Yang , X. Yao , Small 2016, 12, 1295. 26753802 10.1002/smll.201503305

[14] H.‐J. Noh , S. Youn , C. S. Yoon , Y.‐K. Sun , J. Power Sources 2013, 233, 121.

[15] F. Fu , G.‐L. Xu , Q. Wang , Y.‐P. Deng , X. Li , J.‐T. Li , L. Huang , S.‐G. Sun , J. Mater. Chem. A 2013, 1, 3860.

[16] J. Li , C. Cao , X. Xu , Y. Zhu , R. Yao , J. Mater. Chem. A 2013, 1, 11848.

[17] J. Li , W S. Xiong , Y. Liu , Z. Ju , Y. Qian , Nano Energy 2013, 2, 1249.

[18] W. Liu , P. Oh , X. Liu , M.‐J. Lee , W. Cho , S. Chae , Y. Kim , J. Cho , Angew. Chem. Int. Ed. 2015, 127, 4518. 10.1002/anie.201409262 25801735

[19] Y.‐K. Sun , S.‐T. Myung , M.‐H. Kim , J. Prakash , K. Amine , J. Am. Chem. Soc. 2005, 127, 13411. 16173775 10.1021/ja053675g

[20] L. Wang , Y. Lu , J. Liu , M. Xu , J. Cheng , D. Zhang , J. B. Goodenough , Angew. Chem. Int. Ed. 2013, 52, 1964. 10.1002/anie.201206854 23319239

[21] J.‐H. Wang , Y. Wang , Y.‐Z. Guo , Z.‐Y. Ren , C.‐W. Liu , J. Mater. Chem. A 2013, 1, 4879.

[22] Z. Wu , X. Han , J. Zheng , Y. Wei , R. Qiao , F. Shen , J. Dai , L. Hu , K. Xu , Y. Lin , Nano Lett. 2014, 14, 4700. 24979204 10.1021/nl5018139

[23] M. Jo , M. Noh , P. Oh , Y. Kim , J. Cho , Adv. Energy Mater. 2014, 4, 1301583.

[24] Y. Wei , J. Zheng , S. Cui , X. Song , Y. Su , W. Deng , Z. Wu , X. Wang , W. Wang , M. Rao , Y. Lin , C. Wang , K. Amine , F. Pan , J. Am. Chem. Soc. 2015, 137, 8364. 26098282 10.1021/jacs.5b04040

[25] a) J.‐Y. Liao , A. Manthiram , J. Power Sources 2015, 282, 429;

