# Supplementary material for: Multishelled Ni‐Rich Li(NixCoyMnz)O2 Hollow Fibers with Low Cation Mixing as High‐Performance Cathode Materials for Li‐Ion Batteries
Source: Adv Sci (Weinh). 2016 Sep 7;4(1):1600262. doi: 10.1002/advs.201600262 (PMC5238738; doi:10.1002/advs.201600262)
Supplement: Supplementary file 1 — Supplementary [file ADVS-4-0-s001.pdf]

Copyright WILEY-VCH Verlag GmbH & Co. KGaA, 69469 Weinheim, Germany, 2016.

Supporting Information

for *Adv. Sci.,* DOI: 10.1002/advs.201600262

Multishelled Ni-Rich $Li(Ni_xCo_yMn_z)O_2$ Hollow Fibers with Low Cation Mixing as High-Performance Cathode Materials for Li-Ion Batteries

Yihui Zou, Xianfeng Yang, Chunxiao Lv, Tongchao Liu, Yanzhi Xia, Lu Shang, Geoffrey I. N. Waterhouse, Dongjiang Yang, and Tierui Zhang**

Supporting Information

Multi-Shelled Ni-rich $Li(Ni_xCo_yMn_z)O_2$ Hollow Fibers with Low Cation Mixing as High-Performance Cathode Materials for Li-Ion Batteries

Yihui Zou, Xianfeng Yang, Chunxiao Lv, Tongchao Liu, Yanzhi Xia, Lu Shang, Geoffrey I.N. Waterhouse, Dongjiang Yang and Tierui Zhang**

Chemicals: Sodium alginate was supplied by the Bright Moon Seaweed Group (Qingdao, P. R. China). Anhydrous calcium chloride ($CaCl_2$), cobalt acetate tetrahydrate ($Co(CH_3COO)_2 \cdot 4H_2O$), nickel acetate tetrahydrate ($Ni(CH_3COO)_2 \cdot 4H_2O$), manganese acetate tetrahydrate ($Mn(CH_3COO)_2 \cdot 4H_2O$) and lithium carbonate (Li_2CO_3) were purchased from Sinopharm Chemical Reagent (Shanghai, China) and used without further purification. Ethanol and hydrochloric acid were purchased from the Shuangshuang Chemistry Company (Yantai, P. R. China).

Synthesis of multi-shelled $Li(Ni_xCo_yMn_z)O_2$ hollow fibers: A 5.0 wt % aqueous sodium alginate (SA) solution was introduced via a spinneret orifice into a coagulating bath containing a 4.5 wt % aqueous $CaCl_2$ solution to form calcium alginate fibres (Ca-AF). The Ca-AF were subsequently collected and then soaked with vibration in a 1 M HCl solution at 60 °C for 4 h to exchange the Ca^{2+} with H^+. The obtained hydrogen alginate fibres (H-AF) were then soaked in a mixed aqueous solution containing $Ni(CH_3COO)_2 \cdot 4H_2O$, $Co(CH_3COO)_2 \cdot 4H_2O$ and $Mn(CH_3COO)_2 \cdot 4H_2O$ at room temperature for 1 h, yielding metal alginate fibres (M-AF, where M = Ni, Co and Mn). The molar ratios of $Ni^{2+}/Co^{2+}/Mn^{2+}$ in the M-AF were systematically varied, as summarized in Table S1. The M-AF (1 g) were then dipped into a suspension of Li_2CO_3 (2 g) in H_2O:EtOH (1:2, 200 mL) for 0.5 h to obtain lithium-metal alginate fibres (Li-M-AF). The Li-M-AF were pre-oxidized at 500 °C for 2 h, followed by calcination at various temperatures for 8 h to obtain multi-shelled $Li(Ni_xCo_yMn_z)O_2$ hollow fibres.

Characterization: The crystal structures of the products were characterized by powder X-ray diffraction (XRD, DX2700, China), using a diffractometer equipped with Cu Kα X-ray source operating at an accelerating voltage of 40 kV and an applied current of 30 mA. Powder patterns were collected at a scan rate of 2° min^{-1} over the 2θ range 5-90°. The chemical composition of the samples was determined by inductive coupled plasma atomic emission

spectrometry (ICP-AES). The morphology of the $Li(Ni_xCo_yMn_z)O_2$ hollow fibers were investigated by field emission scanning electron microscopy (FESEM; JSM-7001F, JEOL, Tokyo, Japan). TEM and high-resolution TEM (HRTEM) images were obtained using a FEI Tecnai 20 TEM operating at an accelerating voltage of 200 kV. Near surface region chemical compositions were determined by X-ray photoelectron spectroscopy (XPS) using an ESCALab250 electron spectrometer (Thermo Scientic Corporation) equipped with a monochromated Al Kα X-ray source (1486.7 eV, 150 W).

Electrochemical measurements: The $Li(Ni_xCo_yMn_z)O_2$ hollow fibers were mixed with acetylene black, poly(-vinylidene fluoride) (PVDF) and N-methyl-2-pyrrolidone (NMP) in a weight ratio of 8:1:1 to form a slurry. The resultant slurry was then uniformly spread on one side of an aluminium sheet electrode. The active material loading density of the electrode is ~0.92 mg cm^{-2}. The coated aluminium sheet electrode was then dried in a vacuum oven at 120 °C overnight to remove any solvent. The electrolyte for the electrochemical measurements consisted of a solution of 1 M $LiPF_6$ in ethylene carbonate (EC)-dimethyl carbonate (DMC)-diethyl carbonate (DEC) (1:1:1 by weight). CR2016-type coin cells were assembled in a glove box using Li metal as the anode. The discharge and charge measurements were conducted using a cell testing instrument (LAND CT2001A) over the potential range of 2.5 to 4.6 V. Electrochemical impedance spectroscopy (EIS) and Cyclic voltammetry (CV) were performed using an Auto-lab83710 electrochemical workstation. The CV measurement was carried out at a scanning rate of 0.1 mV s^{-1} between 2.5 to 4.6 V. The EIS measurement was performed over a frequency range of 100 kHz to 10 mHz with an applied amplitude of 5 mV. The EIS plots were fitted using the Zsimpwin 3.1 program. The GITT method to measure the Li^+ ion diffusion coefficient was conducted using LAND CT2001A discharged at 0.05 C for 15 min, and rested for 45 min repeatedly from 4.5 V to 2.7 V at 25 °C.

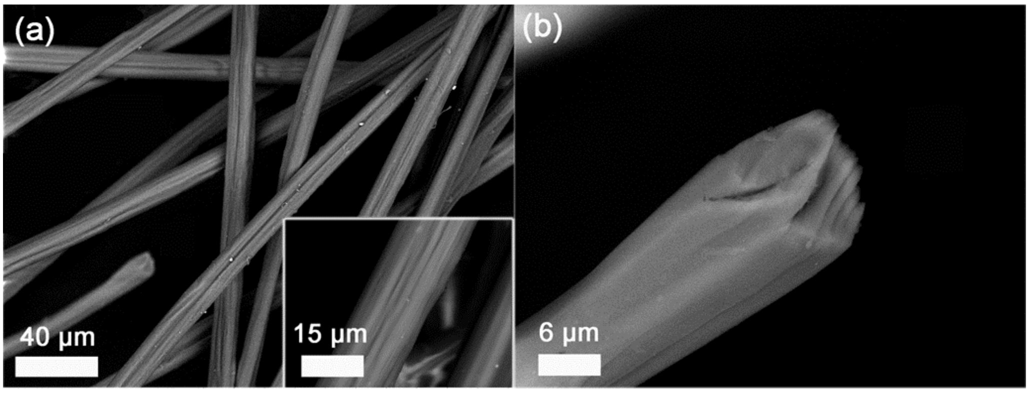

Figure S1. SEM images of calcium alginate fibres (Ca-AF).

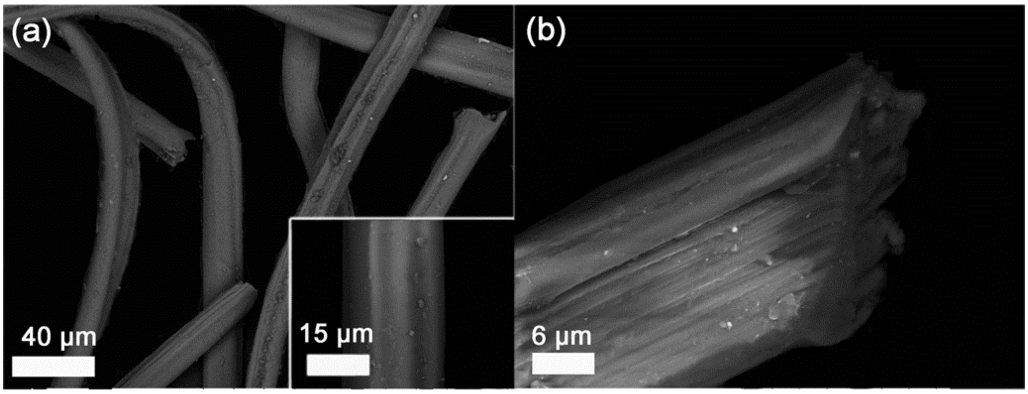

Figure S2. SEM images of lithium-metal alginate fibres (Li-M-AF).

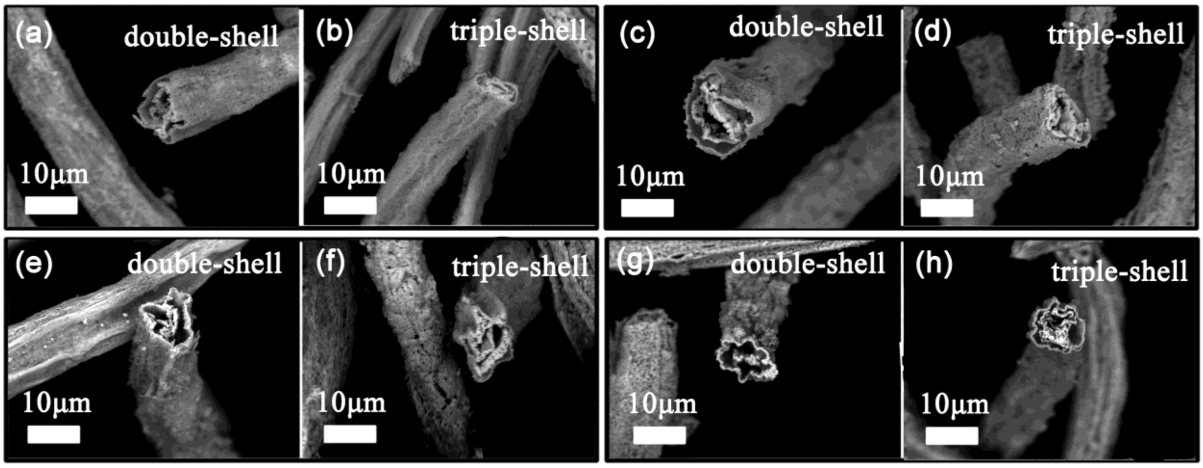

Figure S3. SEM images of multi-shelled $Li(Ni_xCo_yMn_z)O_2$ hollow fibres. (a-b) x = 0.5, (c-d) x = 0.65, (e-f) x = 0.7, and (g-h) x = 0.8.

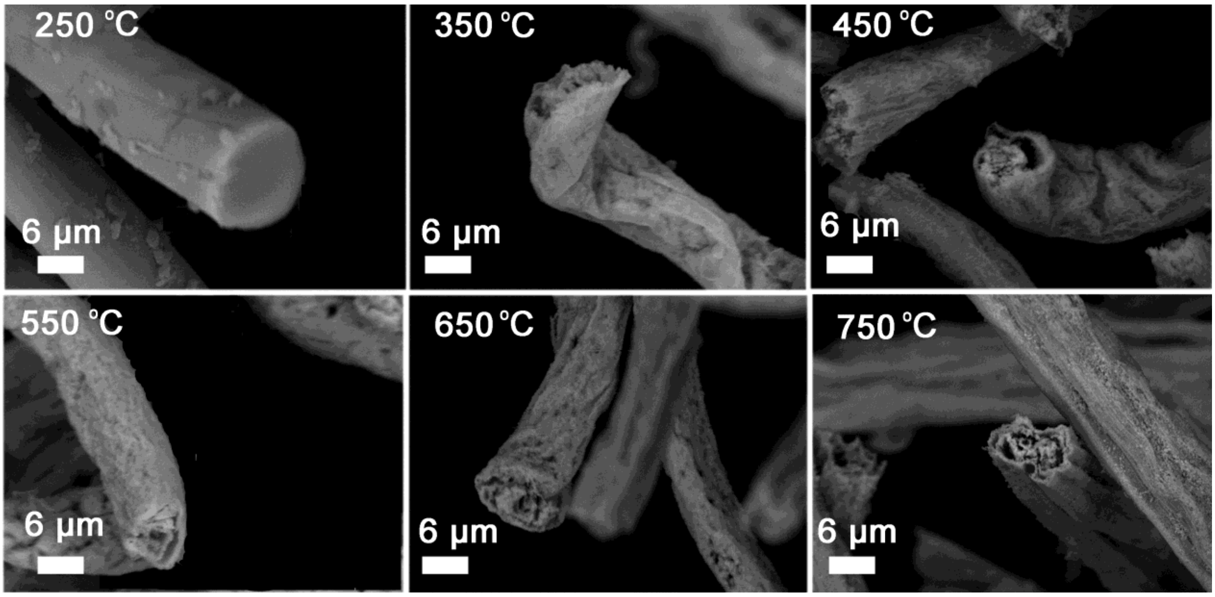

Figure S4. SEM images of the multi-shelled $Li(Ni_{0.65}Co_{0.25}Mn_{0.1})O_2$ hollow fibres annealed at different temperatures for 2 h.

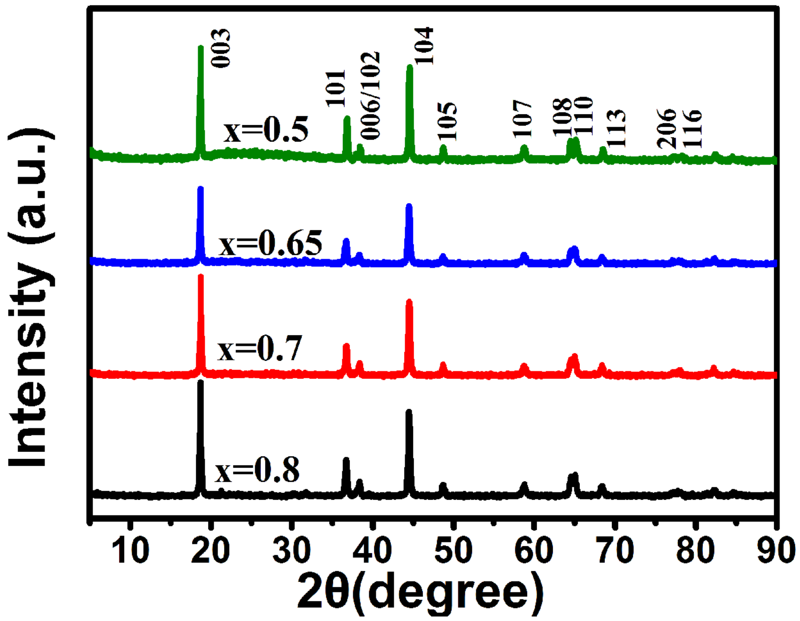

Figure S5. XRD patterns of the multi-shelled $Li(Ni_xCo_yMn_z)O_2$ hollow fibres (x = 0.8, 0.7, 0.65 and 0.5).

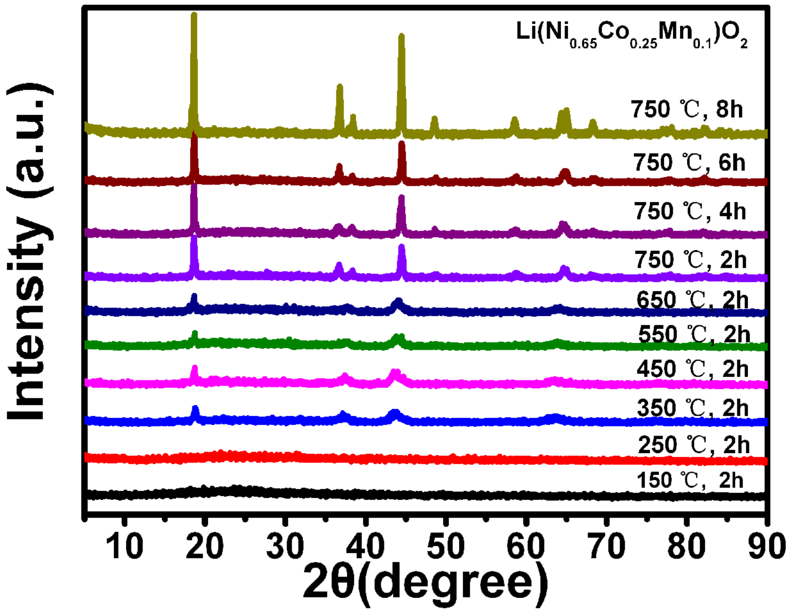

Figure S6. XRD patterns of the multi-shelled Li($Ni_{0.65}Co_{0.25}Mn_{0.1}$)$O_2$ hollow fibres annealed at different temperatures.

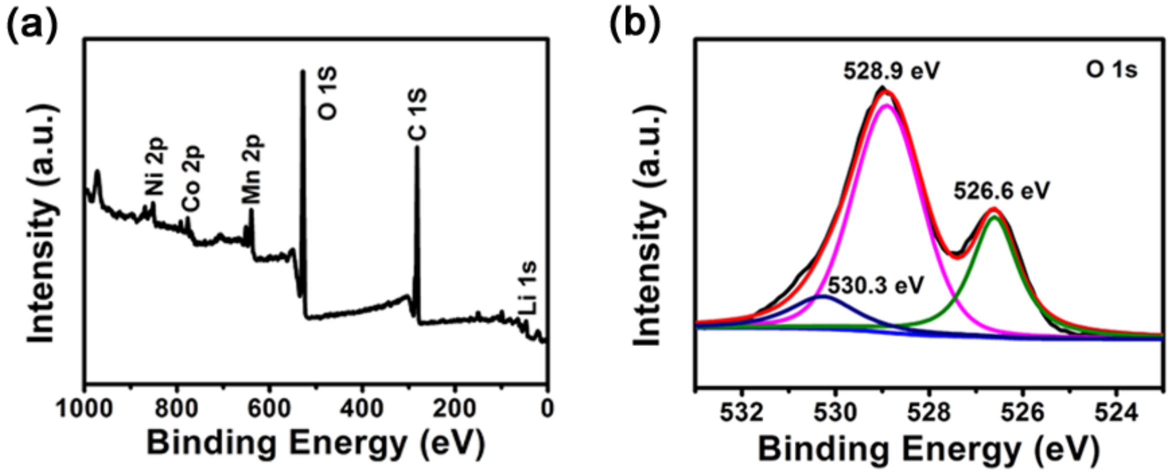

Figure S7. (a) Survey XPS spectrum of Li($Ni_{0.65}Co_{0.25}Mn_{0.1}$)$O_2$ hollow fibres, and (b) corresponding O 1s XPS spectrum.

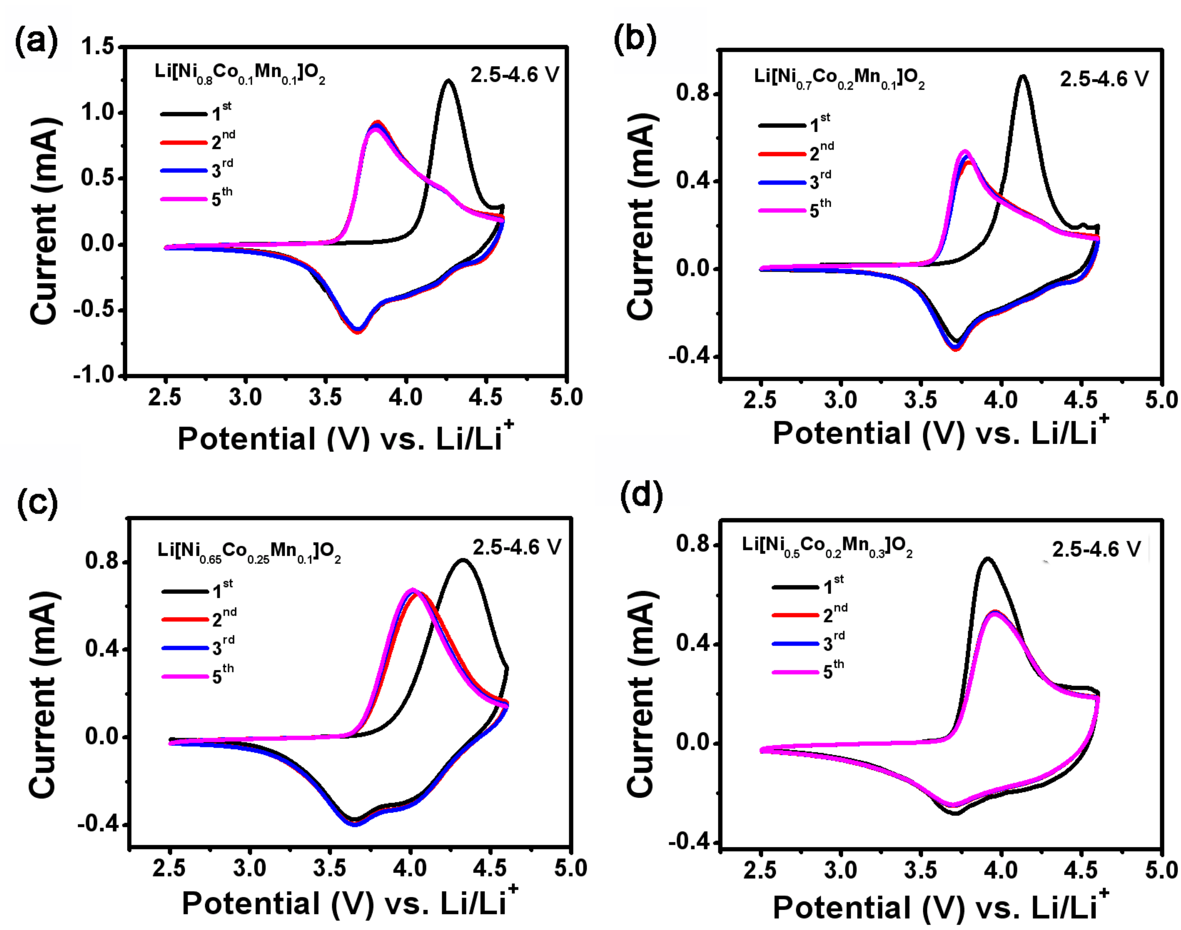

Figure S8. Cyclic voltammetry curves of multi-shelled $Li(Ni_xCo_yMn_z)O_2$ hollow fibres (x = 0.8, 0.7, 0.65 and 0.5) at a scan rate of 0.5 mV s^{-1} in the voltage range 2.5-4.6 V versus Li/Li^+.

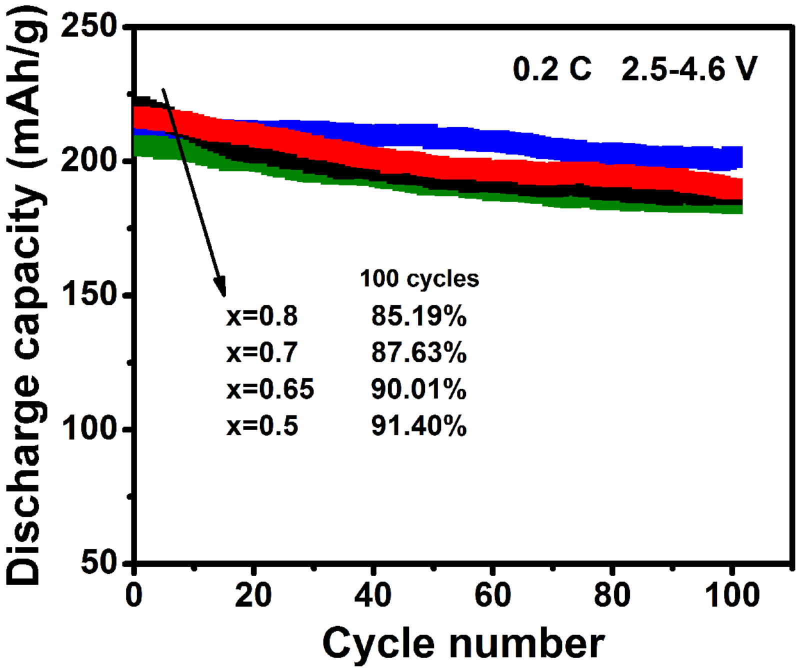

Figure S9. Cycle performance of multi-shelled Li($Ni_xCo_yMn_z$)O_2 hollow fibres (x = 0.8, 0.7, 0.65 and 0.5) at the rate of 0.2 C between 2.5-4.6 V.

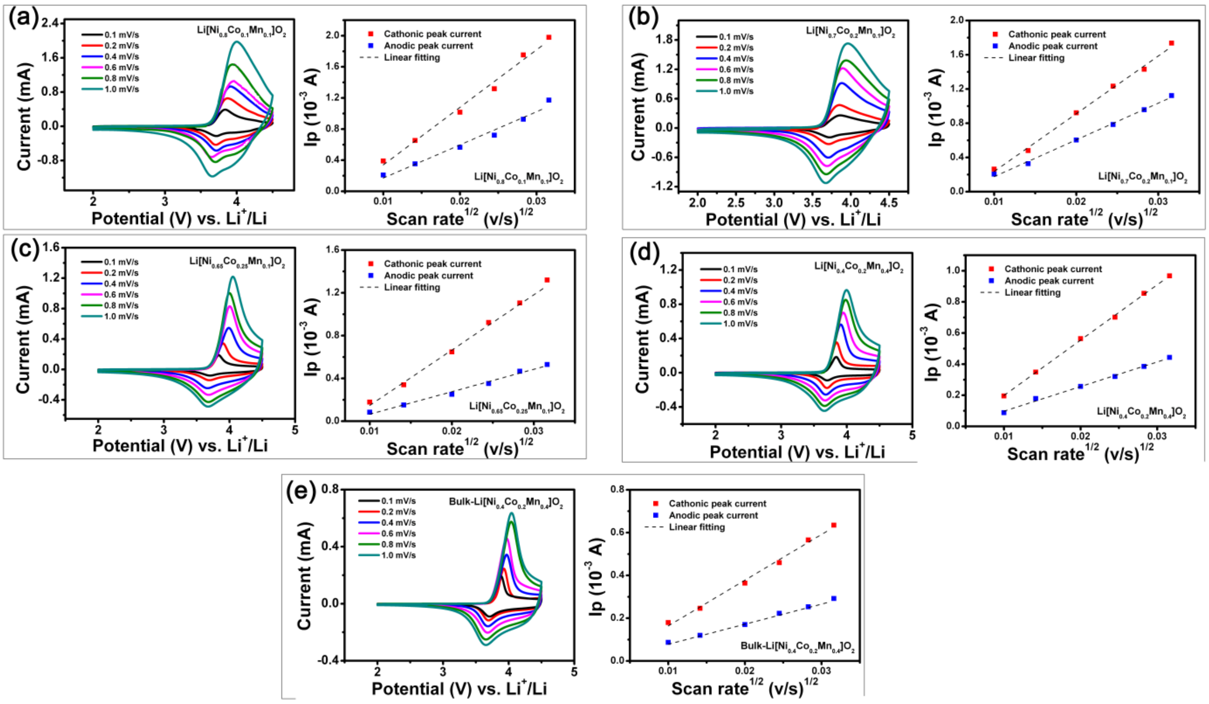

Figure S10. Cycle voltammograms and peak current I_p as a function of square root of scan rate $v^{1/2}$ for different multi-shelled Li($Ni_xCo_yMn_z$)O_2 hollow fibres (a) x = 0.8 (b) x = 0.7 (c) x = 0.65 (d) x = 0.5 and (e) bulk-Li($Ni_{0.5}Co_{0.2}Mn_{0.3}$)O_2.

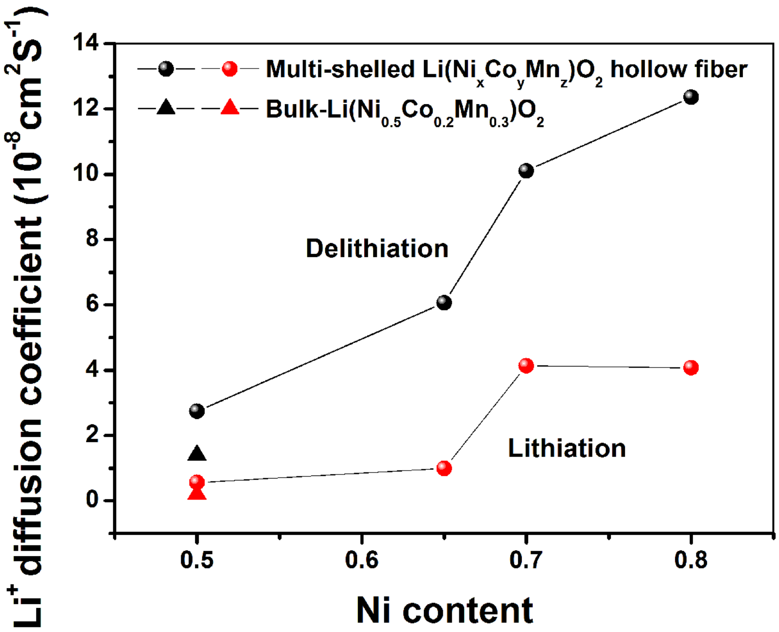

Figure S11. Li$^+$ diffusion coefficient of the multi-shelled Li($Ni_xCo_yMn_z$)O_2 hollow fibres (x = 0.8, 0.7, 0.65 and 0.5) and bulk-Li($Ni_{0.5}Co_{0.2}Mn_{0.3}$)O_2.

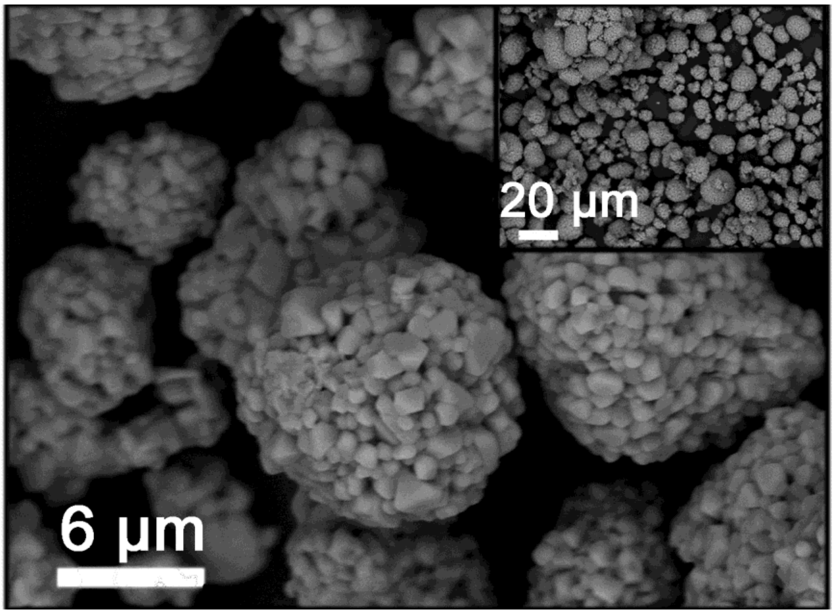

Figure S12. SEM image of bulk-Li($Ni_{0.5}Co_{0.2}Mn_{0.3}$)O_2.

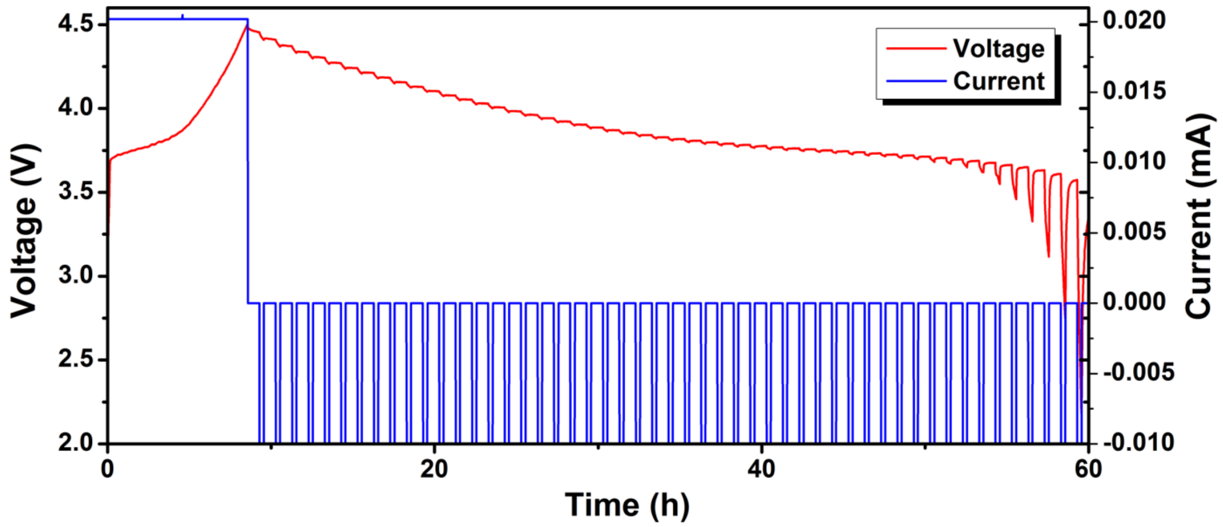

Figure S13. GITT data for a bulk-Li($Ni_{0.5}Co_{0.2}Mn_{0.3}$)O_2 coin cell. The cell was discharged at 0.05 C for 15 minutes, and rest for 45 minutes at room temperature (25 °C) repeatedly until the valtage was less than 2.5 V.

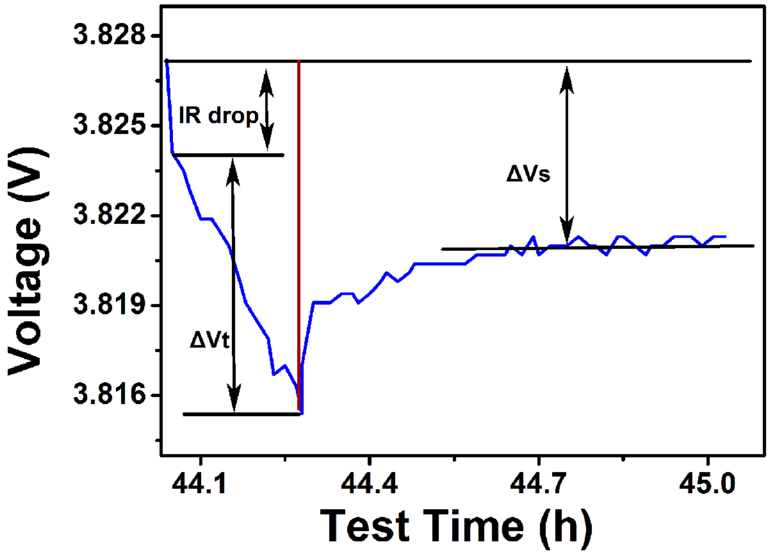

Figure S14. Zoomed-in voltage versus time plot corresponding to that in **Figure S13** when SOC = 0.5. The red line divides the figure into two parts. The left part shows the discharge pulse, whilst the right part shows the rest step.

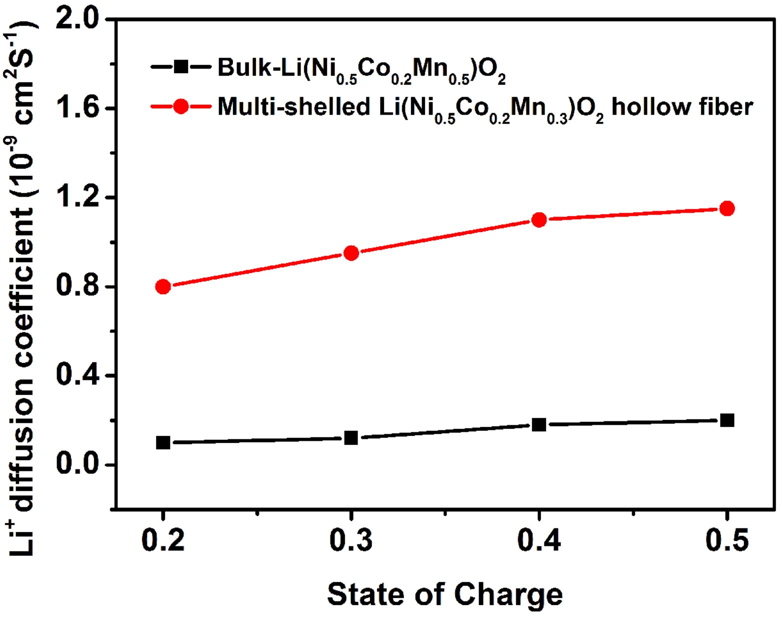

Figure S15. Calculated Li^+ diffusion coefficient at different states of charge (SOC).

Table S1. The molar concentration ratios of Ni^{2+}, Co^{2+} and Mn^{2+} used in the preparation of M-AFs.

sample	$Li(Ni_{0.5}Co_{0.2}Mn_{0.3})O_2$	$Li(Ni_{0.65}Co_{0.25}Mn_{0.1})O_2$	$Li(Ni_{0.7}Co_{0.2}Mn_{0.1})O_2$	$Li(Ni_{0.8}Co_{0.1}Mn_{0.1})O_2$
C_{Ni}^{2+}	0.10 mol L^{-1}	0.15 mol L^{-1}	0.25 mol L^{-1}	0.30 mol L^{-1}
C_{Co}^{2+}	0.05 mol L^{-1}	0.05 mol L^{-1}	0.05 mol L^{-1}	0.05 mol L^{-1}
C_{Mn}^{2+}	0.075 mol L^{-1}	0.03 mol L^{-1}	0.03 mol L^{-1}	0.05 mol L^{-1}

Table S2. Nominal and actual chemical composition of the prepared $Li(Ni_xCo_yMn_z)O_2$ hollow fibres.

Nominal composition of samples	Actual metal composition of samples determined by ICP-AES			
	Li	Ni	Co	Mn
$Li(Ni_{0.5}Co_{0.2}Mn_{0.3})O_2$	1.010	0.503	0.199	0.298
$Li(Ni_{0.65}Co_{0.25}Mn_{0.1})O_2$	0.998	0.651	0.246	0.103
$Li(Ni_{0.7}Co_{0.2}Mn_{0.1})O_2$	1.091	0.702	0.197	0.101
$Li(Ni_{0.8}Co_{0.1}Mn_{0.1})O_2$	1.013	0.803	0.099	0.098

Table S3. Intensity ratio $I_{(003)}/I_{(104)}$ of multi-shelled $Li(Ni_xCo_yMn_z)O_2$ hollow fibres (x = 0.5, 0.65, 0.7 and 0.8).

samples	$Li(Ni_{0.5}Co_{0.2}Mn_{0.3})O_2$	$Li(Ni_{0.65}Co_{0.25}Mn_{0.1})O_2$	$Li(Ni_{0.7}Co_{0.2}Mn_{0.1})O_2$	$Li(Ni_{0.8}Co_{0.1}Mn_{0.1})O_2$
$I_{(003)}/I_{(104)}$	1.31	1.26	1.25	1.24

Table S4. Intensity ratio $I_{(003)}/I_{(104)}$ for multi-shelled $Li(Ni_{0.65}Co_{0.25}Mn_{0.1})O_2$ hollow fibres from 350 °C to 750 °C.

samples	350 °C 2h	450 °C 2h	550 °C 2h	650 °C 2h	750 °C 2h	750 °C 4h	750 °C 6h	750 °C 8h
$I_{(003)}/I_{(104)}$	1.310	1.302	1.304	1.31	1.297	1.285	1.273	1.262

Note S1: The Li^+ diffusion coefficient shown in **Figure S11** is calculated from the Randles-Sevcik equation, which assumes that the apparent D values are constant in the whole phase transition regime and the system is regarded as a homogeneous system:

$$Ip = 2.69\times10^5 n^{3/2} A D_{Li^+} C_{Li^+}{}^{1/2} \nu^{1/2} (25\ °C) \qquad (1)$$

In the equation, *Ip* is the peak current (A), *n* is the number of electrons per reaction species, which is 1 for Li^+, *A* is the electrode area (cm^2), *D* is the diffusion coefficient ($cm^2\ s^{-1}$) for the Li^+ in the electrolyte, ν is the scan rate ($V\ s^{-1}$), *C* is the bulk concentration of Li^+ ion in the electrode ($mol\ cm^{-3}$). The linear relationship of Ip and $v^{1/2}$ is shown in **Figure S10**.

GITT method used to determine the diffusion coefficient of Li^+ ion is as follows :

The cell is subjected to a small constant current, and the potential changes are measured as a function of time. One important assumption is that the diffusion in solid solution electrode (multi-shelled $Li(Ni_{0.5}Co_{0.2}Mn_{0.3})O_2$ HF and commercial bulk $Li(Ni_{0.5}Co_{0.2}Mn_{0.3})O_2$) is one-dimensional diffusion. And we do not take the ohmic potential drop, double-layer charging, charge-transfer kinetics and phase transformation into consideration. So the Li^+ ion diffusion coefficient can be calculated by Fick's law through the following equation.The GITT method to calculation of Li-ion diffusion coefficient is based on the Fick's law through the following equation.

$$Ds = \frac{4}{\pi}\left(\frac{IV_M}{z_A FS}\right)\left[\frac{\left(dV(x)/dx\right)}{dV(t)/d\sqrt{t}}\right]^2 \left(t \langle\langle L^2/D_s\right) \qquad (2)$$

Where Z_A is the charge number of the active material (here is 1 for Li-ion batteries), *F* is the Faraday constant (96485.3 C/mol), *S* (cm^2) is the interfacial area between the electrode and the electrolyte of the cell, *I* (A) is the applied current which is constant during the current pulse, and V_M (cm^3/mol) is the molar volume of the active material. And we can measure the value of dV(t)/d$\sqrt{t}$ and dV(x)/dx by plotting the voltage against the square root of the time during constant current pulse and the equilibrium electrode voltage against the State of Charge (SOC) after each current pulse, respectively. For the multi-shelled $Li(Ni_{0.5}Co_{0.2}Mn_{0.3})O_2$ HF and commercial bulk $Li(Ni_{0.5}Co_{0.2}Mn_{0.3})O_2$, we assume that the solid phase is consisted of spherical partical with radius Rs. Thus, we get Eq.3:

$$Ds = \frac{4}{\pi\tau}\left(\frac{Rs}{3}\right)^2\left(\frac{\Delta Vs}{\Delta Vt}\right)^2 \qquad (3)$$

Where *τ (s)* is the time duration of the pulse. ΔVs and ΔVt can be calculated in **Figure S14** and the inset of **Figure 5b.** Here, we calculated the ΔVs and ΔVt for SOC = 0.2, 0.3, 0.4, 0.5. **Figure S14** and **Figure 5b** shows the ΔVs and ΔVt for SOC = 0.5. And the calculated results were shown in **Figure S15.**
